# Older people in Sweden increasingly enter long-term care with extensive care needs–a register study of first-time users based on the SNAC Stockholm Eldercare study

**DOI:** 10.1007/s10433-025-00906-5

**Published:** 2026-01-13

**Authors:** Bettina Meinow, Ida Goliath, Sarah Wallcook, Maria Flink, Pernilla Alencar Siljehag, Charlotte Klinga, Helena Strehlenert, Åsa von Berens

**Affiliations:** 1 Stockholm Gerontology Research Center, Stockholm, Sweden; 2 Aging Research Center, Department of Neurobiology, Care Sciences and Society, Karolinska Institutet & Stockholm University, Stockholm, Sweden; 3 Division of Nursing, Department of Neurobiology, Care Sciences and Society, Karolinska Institutet, Stockholm, Sweden; 4 Division of Family Medicine and Primary Care, Department of Neurobiology, Care Sciences and Society, Karolinska Institutet, Stockholm, Sweden; 5 Stockholm Research and Development Unit for Older Persons (FOU Nu), Region Stockholm, Sweden; 6 Department of Learning, Informatics, Management and Ethics, Karolinska Institutet, Stockholm, Sweden

**Keywords:** Sweden, LTC entry, LTC utilisation, Older adults, ADL

## Abstract

Although previous studies have addressed factors associated with *current* long-term care (LTC) use, little is known about older adults’ care needs and life situation at the *point of entry* into formal LTC (e.g., home care, institutional care). Using data from the SNAC Stockholm Eldercare study, we identified all individuals aged ≥ 65 in the municipality of Stockholm who entered publicly funded LTC for the first time between 2015 and 2022 (n = 33,393). Descriptive statistics and regression models were used to examine: (1) temporal trends in sociodemographic characteristics and care needs among individuals entering LTC between 2015 and 2022; (2) factors associated with entering LTC with a high level of dependency; and (3) the type and amount of LTC granted.

Sociodemographic characteristics remained largely stable over time, but the proportion entering LTC with high dependency increased from 21% in 2015 to 29% in 2022, suggesting a shift toward more extensive care needs at admission. Higher age (≥ 90), cohabiting, lower income, and at least weekly informal care significantly increased the likelihood of entering LTC with extensive needs. Most first-time users were granted home care (85–87%), although the predicted number of monthly hours slightly declined from 27 to 25, adjusted for sociodemographic and need-related factors. Entry into LTC reflects an interplay of care needs, care-seeking-behaviour, and policies and eligibility criteria. Our findings highlight the importance of further research into how attitudes and beliefs shape care-seeking behaviour, and whether earlier entry into LTC during functional decline could help prevent the development of more extensive needs.

**Supplementary Information:**

The online version contains supplementary material available at 10.1007/s10433-025-00906-5.

## Introduction

Provision of long-term care (LTC) is an increasingly urgent policy concern as many countries face a growing population of older adults in need of support. By international comparison, Sweden has a comprehensive and universal LTC system, primarily funded through local taxation and accessed by all societal groups. Despite retrenchment following the 1990s economic recession (Szebehely and Meagher 2018), the system remains characterised by generous provision (European Commission 2018), with most older adults receiving publicly funded LTC (hereafter “LTC”) during their final years of life (Meinow et al. 2020b).

Entry into LTC marks a transition from managing daily life through personal means – whether independently, with informal caregiving, or privately financed practical assistance – to seeking and receiving support provided by the welfare system. While prior research has focused on determinants of current LTC use (Burrell et al. 2023; Dupraz et al. 2020; Ilinca et al. 2022; Stroh et al. 2022), few studies have examined characteristics associated with *entering* LTC. Understanding the evolving characteristics of first-time LTC users is essential for policy and planning, enabling better service targeting and preventive strategies. This study uses administrative data from Sweden to examine the life circumstances and services granted to older adults entering LTC for the first time, and how these factors changed between 2015 and 2022.

LTC in Sweden is regulated by the Social Services Act (Ministry of Health and Social Affairs 2001: 453), a goal-oriented framework law which guarantees a general right to assistance without specifying detailed entitlements. Municipalities are legally obliged to provide home care in ordinary housing and around-the-clock care in institutional care facilities, the main forms of LTC. Eligibility is based on difficulties managing daily activities, regardless of medical diagnosis. All individuals aged ≥ 65 who perceive a need for assistance with daily tasks, such as household chores and/or personal care (e.g., showering, dressing, toileting) may apply for LTC. Following application, municipal needs assessors (MNAs) conduct needs assessments to determine the type and amount of LTC services. Given the high degree of municipal autonomy, eligibility criteria vary. Under the aging-in-place policy, institutional care is granted only when home-based support is deemed insufficient (Ministry of Health and Social Affairs 2001: 453). LTC user fees are generally low and determined by individual income, with exemptions available (Schön and Heap 2018). Family or household financial resources are not considered.

Numerous studies have examined LTC use through Andersen’s Behavioural Model (Andersen and Newman 1973; Andersen 1995), which categorizes determinants as need, predisposing, and enabling factors (Lederle et al. 2021). Associations have been found with needs-related factors (Brändström et al. 2022; Dupraz et al. 2020; Sm-Rahman et al. 2023; Vlachantoni et al. 2015), as well as predisposing and enabling characteristics, (Brändström et al. 2022; Burrell et al. 2023; Mah et al. 2021; Sm-Rahman et al. 2023; Steinbeisser et al. 2018; Stroh et al. 2022). However, comparability and generalizability of findings are limited by heterogeneity in study designs, populations, societal contexts, explanatory variables, and statistical methodologies. Specifically, in studies lacking key need indicators (e.g., functional impairments and disabilities), predisposing (e.g. age, gender) and enabling factors (e.g., household composition) may appear more influential (Stroh et al. 2022). Moreover, most research addresses current LTC use, although conditions may have evolved after entry (Slobbe et al. 2017).

Evidence on decisive circumstances surrounding LTC entry remains limited. A study from northern Sweden found that most individuals began with home care services corresponding to mild dependency, with intensity increasing soon after (Zingmark and Norström 2021). In the Netherlands, need, age (predisposing), household size and homeownership (enabling) predicted future LTC use (Slobbe et al. 2017), while a German study identified age as the primary driver of transition from no LTC to formal care (Steinbeisser et al. 2018). Despite these insights, little is known about the characteristics and life circumstances of those who *enter* LTC in the Swedish context, and whether these characteristics have changed over time.

**Fig. 1:**
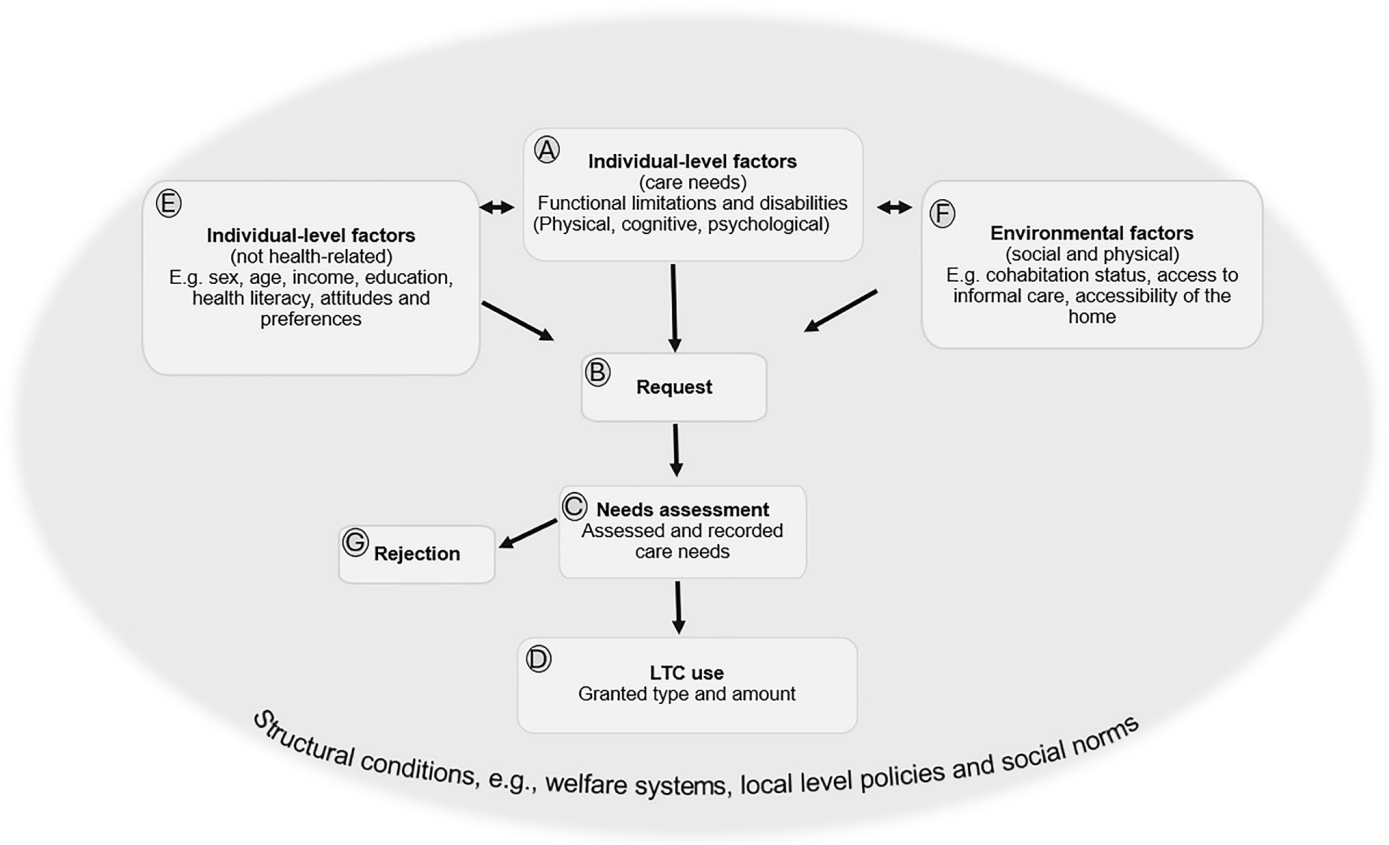
Framework of factors associated with the use of publicly funded long-term care. An adapted version of Andersen’s Behavioural Model

To situate our study within the broader context of care-seeking among older adults, we apply an adapted version of Andersen’s Behavioural Model (Fig. 1). This conceptual framework provides a basis for understanding the progression from emerging care needs to LTC entry and supports interpretation of our empirical findings by delineating key stages (Boxes B–D) and factors that influence these transitions (Boxes A, E, F). Figure 1 conceptualises LTC as a multi-step process: the emergence of care needs (Box A), the individual decision to seek LTC (B), and the outcome of the needs assessment, i.e., whether LTC is granted and, if so, its type and amount (Boxes C, D and G). Individual (predisposing and enabling) factors, including sociodemographic characteristics, attitudes, and knowledge, may influence both functional ability (Box A) and the likelihood of initiating a LTC request (B) (Berglund et al. 2019; Stroh et al. 2022). An individual’s perceived need is a necessary precondition for requesting LTC. However, not all individuals with care needs go on to request LTC. Environmental factors (Box F), such as access to informal care, cohabitation status, and housing conditions, also shape the care-seeking pathway. These factors may determine whether functional limitations and disabilities result in a LTC request or are managed through informal support, home adaptations, and assistive technologies (Brändström et al. 2022; Larsson et al. 2006). For example, living arrangements and caregiver availability may prevent or delay LTC entry (Geerts and Van den Bosch 2012; Steinbeisser et al. 2018), or reduce the amount of care used (von Berens et al. 2025). Conversely, particularly adult children may act as advocates, supporting and facilitating LTC applications on behalf of their relatives (Courbage et al. 2023; Sperber et al. 2014).

Structural conditions operate as a foundational layer shaping both the emergence of care needs, and the care-seeking process. These include welfare systems, cultural norms, and institutional arrangements, which influence not only the organization and delivery of LTC services, but also how care needs emerge, are perceived, and acted upon (Mah et al. 2021). For example, the role of individual and family financial resources in requesting LTC is shaped by systems features such as eligibility criteria and user fees (Lera Torres et al. 2021).

In Sweden, where LTC is publicly funded and needs-based, functional limitations and disabilities have historically been the main determinants of LTC use and service intensity (Brändström et al. 2022; Meinow et al. 2005; von Berens et al. 2025). However, in the aftermath of the 1990s economic recession, the LTC system underwent substantial structural reforms, marked by significant retrenchment in service (Szebehely and Meagher 2018). Despite a nearly 30% increase in the population aged ≥ 80 since 2000, institutional care capacity has declined by over 30%, now serving 10% of this age group (National Board of Health and Welfare 2023). Home care coverage has also declined, from 23 to 17% over the past decade. Notably, these reductions have not coincided with improvements in older adults’ functional ability (Falk Erhag et al. 2021; Johansson and Schön 2017; Meinow et al. 2022). Such structural shifts may affect both the propensity of older adults to initiate LTC requests when care needs arise (Box B) and the outcomes of subsequent needs assessments (Box C and D). For instance, if eligibility thresholds are perceived as more restrictive, individuals may delay seeking care until needs become more extensive. This underscores the embedded nature of structural factors across the care-seeking pathway.

Monitoring shifts in older adults’ life circumstances, functional limitations, and disability is vital for policy development and planning. It also informs ongoing debates on the scope of public responsibility for LTC and promotes a preventive approach to ageing and LTC provision. Using administrative data from the SNAC Stockholm Eldercare Study, we analysed LTC entry based on documentation from municipal needs assessments resulting in granted services, as recorded by MNAs (Boxes C and D).

The objectives of this study were to.

1. examine sociodemographic characteristics, access to informal care and the level of functional limitations and disability among first-time users of LTC and how these changed between 2015 and 2022.
2. address factors that could indicate late entry into LTC by analysing factors associated with entering LTC with an already high level of dependency.
3. analyse how the type and amount of LTC granted to first-time users, relative to their needs and sociodemographic characteristics, changed between 2015 and 2022.

## Method

### Data and study population

We identified first-time users of LTC (n = 33 393) using data from the SNAC Stockholm Eldercare study, part of the Swedish National Study on Ageing and Care (SNAC) (Lagergren et al. 2004). Data comprised municipal register data on all initial LTC grants for individuals aged ≥ 65 in Stockholm from January 1, 2015, to December 31, 2022.

### Variables

Variable selection was informed by the conceptual framework (Fig. 1), focusing on indicators of factors related to LTC use (Boxes A–F) and available in the register data. For individuals who requested and were granted LTC, the data included indicators of individual-level characteristics (Box E), environmental factors (Box F), the MNA’s assessment of care needs (Box C; based on factors in Box A), and the corresponding LTC services granted (Box D). No data were available for non-applicants (Box B) or for individuals whose applications were denied (Box G).

### LTC use: granted type and amount

Data on LTC use included three binary variables indicating use of home care alone or in combination with other home-based services (e.g., day care, accompaniment services) (yes/no), only other types of home-based services (yes/no), and institutional care (yes/no). Institutional care encompassed 24-h services in residential care facilities. The amount of home care was measured in monthly hours and treated as a continuous variable.

### Individual-level factors

Age was calculated based on the date of birth and the date of first LTC grant and categorized into 10-year intervals, with the oldest group defined as ≥ 90 years (categorical). Gender was categorized as a binary variable (female/male). Annual personal income, including pension and property income, was stratified into quartiles for the corresponding year (categorical). These sociodemographic indicators were obtained by MNAs from population registers. Country of birth was assessed by the MNAs as a binary variable, indicating whether the individual was born in a Nordic country (yes/no).

### Environmental factors

Cohabitation status was recorded binary as cohabiting or living alone. Frequency of informal care, i.e., unpaid care from family, friends, or non-professionals, was categorized by MNAs as never, rarely, or at least once a week. For individuals granted home care, cohabitation with a partner already receiving home care was recorded as a binary variable (yes/no).

### Assessed care needs

Categorical indicators of care needs were assessed by MNAs, covering dependency in personal activities of daily living (ADL), physical and cognitive functional impairments, as well as mental health. ADL needs were measured using the Katz index (Katz 1983), with dependence in each of the five tasks (showering, dressing, toileting, indoor mobility, and eating) scored binarily as dependent or independent. A total ADL dependency score (range 0–5) was summed. Mobility limitations were categorised as: none, slight (able to walk outdoors with assistive devices and manage small stairs); moderate (able to move indoors with assistive devices unable to manage stairs or go outdoors); or severe (dependent on personal assistance or wheelchair use, unable to manage independently). Cognitive impairment was assessed using a shortened version of the Berger scale (Berger 1980) and categorized as: no memory problems (Berger 0); slight (occasional disorientation/confusion, Berger 1); severe (frequent disorientation/confusion, Berger 2–4); or very severe (complete disorientation and memory loss, Berger 5–6). Reported feelings of worry and insecurity were categorized as none, mild, or severe/very severe.

### Analyses

We conducted analyses to examine characteristics of first-time LTC users, factors linked to late entry into LTC, and services granted. Changes in individual-level characteristics, the social environment and assessed care needs from 2015 to 2022 were examined using descriptive statistics stratified by year. Linear time trends were tested with unadjusted bivariate analyses, calculating p-values based on logistic regression for categorical variables and linear regression for age. In these analyses, year was treated as a continuous independent variable, while sociodemographic and need-related variables served as dependent outcomes.

To analyse factors linked to LTC entry with high dependency, we conducted bivariate and adjusted logistic regressions using three dichotomized indicators of extensive care needs that capture different aspects of physical, cognitive and mental health: 4–5 ADL dependencies, severe/very severe cognitive impairment, and severe/very severe feelings of worry/insecurity. Cutoffs reflect levels requiring substantial daily support. Adjusted models were estimated separately for each need indicator, and all independent variables were included simultaneously (Table 1).

**Table 1:** Characteristics of first-time users of LTC by year

	2015	2016	2017	2018	2019	2020	2021	2022	Total	*P*-value
	n = 3824	n = 4259	n = 4278	n = 4349	n = 4373	n = 3936	n = 4504	n = 3870	n = 33,393	
	%	%	%	%	%	%	%	%	%	
*Individual-level and environmental factors*										
Age										
65–69	14.9	14.4	13.3	14.2	12.8	13.6	12.5	11.2	13.4	< 0.001
70–74	14.5	15.6	15.7	15.8	16.3	14.6	12.8	13.6	14.9	< 0.001
75–79	18.0	17.9	19.3	18.5	19.1	21.0	21.8	23.0	19.8	< 0.001
80–84	21.4	22.3	19.9	20.7	21.4	20.6	22.3	21.3	21.3	0.354
85–89	20.6	19.4	18.9	19.4	18.4	17.7	18.1	18.1	18.8	< 0.001
90 +	10.6	10.4	12.8	11.4	12.1	12.4	12.6	12.8	11.9	< 0.001
Mean/Median	79.5/80	79.4/80	79.7/80	79.4/80	79.6/80	79.6/80	80/80	80.1/80	79.6/80	< 0.001
Born in a Nordic country	85.1	85.4	84.7	87.2	84.3	84.9	84.4	84.2	85.1	0.211
*Gender and cohabitation status*										
Women living alone	43.2	44.4	44.0	44.1	43.6	41.3	42.1	43.5	43.3	0.044
Men living alone	21.0	21.1	21.3	22.6	21.5	24.1	23.0	21.9	22.1	0.011
Women cohabiting	15.5	15.0	15.8	14.6	15.3	14.4	14.7	14.0	14.9	0.031
Men cohabiting	20.3	19.5	18.9	18.7	19.6	20.2	20.2	20.6	19.7	0.222
Lives with a partner who is also granted home care ^b^	9.8	8.8	9.3	9.3	8.6	8.2	8.2	8.4	8.8	0.004
*Frequency of informal care*										
Never	15.5	16.1	16.6	17.5	15.1	16.2	15.7	16.9	16.2	0.459
Seldom	24.8	25.1	26.1	26.5	26.5	24.1	22.9	23.4	25.0	0.028
One or several times/week	29.8	29.0	28.9	28.4	28.3	30.0	30.9	30.0	29.4	0.040
Daily	29.9	29.9	28.3	27.7	30.1	29.7	30.5	29.8	29.5	0.112
***Assessed care needs***										
*Number of ADL dependencies*										
0	46.9	45.8	45.1	45.8	46.8	45.6	42.6	42.6	45.3	< 0.001
1	14.2	14.1	14.6	13.1	13.5	12.0	12.4	12.8	13.4	< 0.001
2–3	18.1	18.1	17.5	17.4	16.6	15.3	16.3	15.7	16.9	< 0.001
4–5	20.9	22.0	22.9	23.8	23.1	27.2	28.7	28.9	24.4	0.003
*Mobility limitations*										
None/slight	66.7	65.7	65.5	64.9	64.0	64.3	61.7	62.6	64.4	< 0.001
Moderate	22.7	22.7	23.2	22.9	24.3	21.4	24.7	24.6	23.3	0.019
Severe	10.6	11.6	11.3	12.2	11.7	14.4	13.6	12.9	12.3	< 0.001
*Cognitive impairment*										
None	64.3	63.7	64.2	63.8	63.2	61.8	60.9	61.5	62.9	0.005
Slight	25.4	25.2	25.7	25.7	26.6	26.9	27.5	27.0	26.2	0.001
Severe/ very severe	10.3	11.1	10.1	10.5	10.2	11.3	11.7	11.5	10.8	0.013
*Feelings of worry/insecurity*										
None	67.0	64.3	65.8	64.2	65.0	64.6	61.6	62.9	64.4	0.001
Slight	23.4	25.3	24.0	25.3	25.2	24.7	27.8	26.6	25.3	< 0.001
Severe/ very severe	9.6	10.4	10.2	10.6	9.8	10.8	10.6	10.6	10.3	< 0.001
*LTC use*										
Institutional care	4.8	5.1	4.6	5.2	4.6	6.3	6.0	5.0	5.2	0.021
Home care	87.1	86.5	87.8	87.2	87.8	85.3	83.7	85.1	86.3	< 0.001
Only other types of services in ordinary housing	8.1	8.4	7.6	7.6	7.7	8.5	10.1	9.9	8.5	< 0.001

^a^*P*-value for linear trend over the years, tested using bivariate linear regression for age and logistic regression for categorical variables. ^b^Information only available for individuals granted home care. Missing information: cohabitation status: n = 735 (2.2%); born in a Nordic country: n = 13,858 (41.5%); lives with a partner who is also granted home care: n = 86 (0.3%); frequency of informal care: n = 6252 (18.7%); number of ADL dependencies: n = 3023 (9.1%); mobility limitations: n = 1943 (5.8%); cognitive impairment: n = 3589 (10.8%); worry/insecurity: n = 5924 (17.7%)

To examine the type of LTC granted in relation to assessed needs, we estimated the probability of institutional versus home care using bivariate and adjusted logistic regression models, including year, individual-level characteristics, the social environment, and indicators of assessed needs as independent variables. We also analysed monthly home care hours in relation to assessed needs and examined changes over time. Due to the skewed distribution of home care hours, Poisson regression models were applied.

To facilitate interpretation, results from the regression analyses are presented as predicted margins (PM) with 95% confidence intervals. Results from adjusted models are presented in Tables 2, 3, 4, while results from bivariate models are presented in the Supplementary file, Tables S1-S3. Predicted margins were estimated for each category of the included variables, while all other covariates were held at their respective reference values. Average marginal effects were estimated to assess statistical differences between reference categories and other categories of the predictor variables.

**Table 2:** Estimated probability of entering LTC with a high level of care needs: ADL dependencies, cognitive impairment, and problems with worry/insecurity. Adjusted models presented as predictive margins

	**4–5 ADL –dependencies (n = 30 370)**			**Severe/very severe cognitive impairment (n = 29 804)**			**Severe/very severe feelings of worry/insecurity (n = 27 469)**		
	PM^a^	95% CI		PM^a^	95% CI		PM^a^	95% CI	
*Year*									
2015 (Ref.)	0.210	0.197;	0.223	0.101	0.092;	0.112	0.095	0.085;	0.105
2016	0.222	0.209;	0.233	0.110	0.101;	0.120	0.102	0.091;	0.111
2017	0.231**	0.219;	0.244	0.102	0.092;	0.111	0.102	0.092;	0.111
2018	0.241***	0.229;	0.254	0.104	0.095;	0.113	0.104	0.095;	0.114
2019	0.232*	0.219;	0.244	0.103	0.094;	0.112	0.098	0.090;	0.108
2020	0.269***	0.255;	0.283	0.113	0.102;	0.123	0.108	0.098;	0.119
2021	0.279***	0.266;	0.292	0.115	0.105;	0.124	0.106	0.097;	0.116
2022	0.289***	0.275;	0.303	0.119**	0.109;	0.129	0.111*	0.100;	0.121
***Individual-level and environmental factors***									
*Age*									
65–69	0.230*	0.220;	0.242	0.095***	0.086;	0.104	0.164***	0.152;	0.176
70–74	0.247	0.235;	0.259	0.099**	0.091;	0.108	0.108	0.099;	0.117
75–79	0.234	0.224;	0.244	0.112	0.105;	0.120	0.096	0.088;	0.103
80–84 (Ref.)	0.247	0.237;	0.257	0.116	0.109;	0.124	0.096	0.088;	0.103
85–89	0.248	0.237;	0.260	0.120	0.111;	0.127	0.090	0.082;	0.097
90 +	0.284***	0.270;	0.297	0.095***	0.086;	0.103	0.076***	0.068;	0.085
*Income*									
Q1	0.284***	0.273;	0.294	0.104					
	0.097;	0.111	0.121***	0.113;	0.129				
Q2	0.256***	0.247;	0.266	0.109	0.102;	0.115	0.105**	0.099;	0.113
Q3	0.240***	0.231;	0.248	0.108	0.102;	0.114	0.092	0.086;	0.099
Q4 (Ref.)	0.211	0.203;	0.220	0.112	0.106;	0.119	0.091	0.084;	0.098
*Born in a Nordic country*									
No (Ref.)	0.232	0.218;	0.248	0.117	0.106;	0.129	0.124	0.112;	0.136
Yes	0.242	0.236;	0.250	0.108	0.102;	0.112	0.099***	0.095;	0.104
Missing	0.256**	0.249;	0.264	0.108	0.102;	0.113	0.102**	0.096;	0.108
*Gender and cohabitation status*									
Women living alone (Ref.)	0.182	0.175;	0.188	0.082	0.077;	0.086	0.106	0.100;	0.111
Men living alone	0.199**	0.189;	0.208	0.091**	0.085;	0.098	0.080***	0.074;	0.087
Women cohabiting	0.329***	0.315;	0.343	0.154***	0.143;	0.164	0.136***	0.123;	0.147
Men cohabiting	0.383***	0.370;	0.396	0.144***	0.136;	0.153	0.101	0.092;	0.110
*Lives with a partner who is also granted home care*									
No (Ref.)	0.239	0.234;	0.244	0.094	0.090;	0.097	0.091	0.087;	0.094
Yes	0.120***	0.109;	0.130	0.051***	0.044;	0.057	0.040***	0.031;	0.046
Missing^b^	0.707***	0.685;	0.730	0.541***	0.516;	0.566	0.455***	0.430;	0.482
*Frequency of informal care*									
Never/seldom (Ref.)	0.190	0.184;	0.200	0.071	0.066;	0.076	0.095	0.089;	0.100
At least once a week	0.284***	0.277;	0.291	0.139***	0.133;	0.144	0.115***	0.109;	0.120
Missing	0.227***	0.215;	0.240	0.075	0.067;	0.095	0.085	0.077;	0.094

^a^Predictive margin (estimated marginal probabilities) The adjusted models were conducted separately for each need indicator variable; all the independent variables (including the other need indicators) were included in the models simultaneously.

^b^Information is not available for individuals granted institutional care. For individuals granted home care services, data is missing for *n* = 86. Ref. = Reference category. Significance levels are based on average marginal effect tests. * *p* ≤ 0.05 ** *p* ≤ 0.01 *** *p* ≤ 0.001

**Table 3:** Estimated probability of being granted institutional care versus home care by year, individual-level and environmental factors and assessed care needs. Adjusted model presented as predictive margins (n = 30 410)

	PM^a^	95% CI	
*Year*			
2015 (Ref.)	0.060	0.053;	0.066
2016	0.055	0.050;	0.061
2017	0.051*	0.046;	0.057
2018	0.054	0.048;	0.060
2019	0.048**	0.043;	0.054
2020	0.050*	0.045;	0.056
2021	0.050*	0.045;	0.055
2022	0.047***	0.041;	0.052
***Individual-level and environmental factors***			
*Age*			
65–69	0.060***	0.054;	0.066
70–79	0.041***	0.038;	0.044
80–89 (Ref.)	0.049	0.046;	0.052
90 +	0.077***	0.071;	0.084
*Born in a nordic country*			
No (Ref.)	0.054	0.047;	0.060
Yes	0.057	0.053;	0.060
Missing	0.046	0.043;	0.049
*Income*			
Quartile 1 (Ref.)	0.056	0.051;	0.060
Quartile 2	0.050	0.046;	0.054
Quartile 3	0.052	0.048;	0.056
Quartile 4	0.049*	0.045;	0.053
*Gender and cohabitation status*			
Women living alone (Ref.)	0.060	0.056;	0.066
Men living alone	0.069**	0.064;	0.074
Women cohabiting	0.031***	0.027;	0.035
Men cohabiting	0.035***	0.031;	0.038
*Frequency of informal care*			
Never/seldom (Ref.)	0.057	0.053;	0.01
At least once a week	0.039***	0.036;	0.041
Missing	0.086***	0.079;	0.093
***Assessed care needs***			
*Number of ADL-dependencies*			
0 (Ref.)	0.012	0.010;	0.015
1	0.023***	0.018;	0.029
2	0.047***	0.039;	0.054
3	0.078***	0.067;	0.089
4	0.071***	0.063;	0.080
5	0.159***	0.142;	0.177
Missing	0.034***	0.026;	0.412
*Mobility limitations*			
None/slight (Ref.)	0.044	0.041;	0.049
Moderate	0.038	0.037;	0.044
Severe	0.082***	0.072;	0.086
Missing	0.056	0.042;	0.069
*Cognitive impairment*			
None (Ref.)	0.025	0.022;	0.027
Slight	0.049***	0.044;	0.052
Severe/very severe	0.171***	0.152;	0.178
Missing	0.039**	0.032;	0.045
*Feelings of worry/insecurity*			
None (Ref.)	0.032	0.029;	0.036
Slight	0.056***	0.051;	0.060
Severe/very severe	0.115***	0.105;	0.124
Missing	0.040**	0.035;	0.045

^a^*PM* = Predictive margins; *Ref*. = Reference category. Significance levels are based on average marginal effect tests. * *p* ≤ 0.05 ** *p* ≤ 0.01 *** *p* ≤ 0.001

**Table 4:** Predicted monthly hours of home care (predicted margins) by year, individual-level and environmental factors and assessed care needs, (n = 28 419). Adjusted model

	PM^a^	95% CI	
*Year*			
2015 (Ref.)	27.13	26.94;	27.32
2016	25.83***	25.65;	26.00
2017	26.35***	26.18;	26.52
2018	25.45***	25.30;	25.62
2019	24.96***	24.80;	25.12
2020	25.23***	25.07;	25.40
2021	25.10***	24.95;	25.26
2022	24.73***	24.56;	24.89
***Individual-level and environmental factors***			
*Age*			
65–69	26.15***	25.98;	26.32
70–79	25.39	25.29;	25.49
80–89 (Ref.)	25.27	25.18;	25.36
90 +	26.33***	26.16;	26.50
*Born in a Nordic country*			
No (Ref.)	26.73	26.51;	26.94
Yes	25.62***	25.54;	25.71
Missing	25.22***	25.13;	25.32
*Income*			
Quartile 1 (Ref.)	25.69	25.56;	25.82
Quartile 2	25.35***	25.23;	25.47
Quartile 3	25.31***	25.19;	25.42
Quartile 4	25.91*	25.78;	26.03
*Gender and cohabitation status*			
Women living alone (Ref.)	28.50	28.87;	29.11
Men living alone	28.96***	29.39;	29.72
Women cohabiting	21.57***	21.32;	21.61
Men cohabiting	21.32***	20.96;	21.20
*Lives with a partner who is also granted home care*			
No (Ref.)	25.61	25.54;	25.67
Yes	25.02***	24.80;	25.24
*Frequency of informal care*			
Never/seldom (Ref.)	25.97	25.83;	26.01
At least once a week	24.77***	24.69;	24.85
Missing	27.80***	27.63;	27.98
***Assessed care needs***			
*Number of ADL-dependencies*			
0 (Ref.)	10.04	9.98;	10.10
1	19.65***	19.50;	19.79
2	32.07***	31.85;	32.28
3	41.45***	41.13;	41.76
4	53.82***	53.52;	54.11
5	59.47***	59.04;	59.90
Missing	22.43***	22.17;	22.69
*Mobility limitations*			
None/slight (Ref.)	22.21	22.47;	22.67
Moderate	25.84***	25.61;	25.84
Severe	34.96***	34.25;	34.68
Missing	22.22	21.86;	22.60
*Cognitive impairment*			
None (Ref.)	24.33	24.25;	24.41
Slight	26.95***	27.56;	27.82
Severe/ very severe	30.63***	30.35;	30.89
Missing	25.34	24.31;	24.78
*Feelings of worry/insecurity*			
None (Ref.)	24.72	24.64;	24.80
Slight	26.95***	26.82;	27.08
Severe/ very severe	28.12***	27.88;	28.36
Missing	25.34***	25.18;	25.51

^a^*PM* = Predictive margins (predicted average hours/month). *Ref*. = reference category. Significance levels are based on average marginal effect tests. * *p* ≤ 0.05 ** *p* ≤ 0.01 *** *p* ≤ 0.001

Because information on country of birth and frequency of informal care was missing for 42% and 19% of respondents, we sought to avoid bias resulting from listwise deletion. To ensure transparency, we included cases with incomplete data and treated missing values as a separate category in regression models, except for cohabitation status (2.2%), which lacked information on all needs-related variables. Consequently, coefficients for missing values in Tables 2–4 reflect systematic differences between respondents with and without missing data, rather than representing substantive effects. Data were analysed using Stata 17.0 for Windows.

## Results

### Characteristics of first-time users of LTC 2015–2022

Analyses for objective 1 showed that between 2015 and 2022, about 4000 individuals per year entered LTC for the first time (Table 1). This corresponds to 28–31 individuals per 1000 inhabitants aged ≥ 65, except in 2020 when rate dropped to 26 per 1000 (Statistics Sweden 2024). Sociodemographic characteristics were largely stable; changes in age and informal care were statistically significant but marginal. The mean/median age was ~ 80 years; ~ 60% were women, two thirds lived alone, and cohabitation declined slightly (9.8% to 8.4%, *p* = 0.004). About 15% were born outside the Nordic countries (with substantial missing data), and ~ 60% received informal care at least weekly, 30% daily.

The proportion needing help with 4–5 ADLs rose from 21 to 29% (*p* = 0.003; Table 1), persisting after adjustment for sociodemographic variables (*p* ≤ 0.001; Table 2). Cognitive impairment among LTC first-time users remained stable; about two-thirds had some impairment, 10% severe (Table 1). Similarly, according to the MNA’s, about 10% experienced severe worry/insecurity. These proportions remained unchanged after controlling for covariates (Table 2).

### Factors associated with a high level of care needs when entering LTC

Addressing objective 2, Table 2 presents the results from the analyses estimating the marginal probabilities of entering LTC with extensive needs, based on three indicators: need for assistance with 4–5 ADLs, severe/very severe cognitive impairment, and severe/very severe worry or feelings of insecurity. Bivariate and adjusted estimates were largely consistent (Table 2, Table S1). The likelihood of entering LTC with ADL dependencies was highest among cohabiting men (0.383; adjusted model), followed by cohabiting women (0.329), and lower among individuals living alone. High age (≥ 90 years), lower income (Q1-Q3), and receiving informal care at least weekly were also associated with higher probabilities of entering LTC with 4–5 ADL dependencies. Severe/very severe cognitive impairment at LTC entry was slightly more common among cohabiting individuals and those receiving informal care at least weakly, but less frequent in the youngest and oldest age groups and among cohabiting partners both receiving home care.

Severe/very severe worry/insecurity was most frequent in ages 65–69 and slightly higher among cohabiting women. Individuals born outside the Nordic countries had a slightly lower probability of entering LTC with extensive care needs related to worry/insecurity. Cohabiting with a partner already receiving home care was consistently associated with lower probabilities of LTC entry with extensive needs across all three need indicators (Table 2).

### Type and amount of LTC granted to first-time users

Analysing granted LTC over the years (objective 3) showed that home care dominated first-time LTC grants (85–87%) throughout the study period (Table 1). Between 8 and 10 percent entered LTC through other forms of home-based support, whereas 4 to 6 percent transitioned directly to institutional care. Adjusted probabilities of institutional care declined from 0.060 to 0.047 between 2015 and 2022 (Table 3). Individuals requiring assistance with all ADLs and those with severe/very severe cognitive impairment exhibited the highest predicted probabilities of being granted institutional care (0.159 and 0.171, respectively). Age ≥ 90, living alone (for both women and men, *p* ≤ 0.001; data not shown), and infrequent informal care were also significantly associated with a higher likelihood of direct transition to institutional care.

Among individuals entering LTC through home care, predicted monthly hours declined slightly from 27 to 25 between 2015 and 2022, adjusted for individual-level characteristics, characteristics of the social environment and assessed care needs (Table 4). ADL showed the strongest association with granted home care hours. Individuals needing assistance with all ADLs received on average 59 h compared to 10 h for those without ADL needs. Mobility limitations, cognitive impairment and worry/insecurity were also linked to more hours. Moreover, individuals living alone were granted more hours than those cohabiting.

## Discussion

While previous research has primarily focused on determinants of *current* LTC use (Burrell et al. 2023; Ilinca et al. 2022; Stroh et al. 2022), this Swedish register-based study contributes a novel perspective by examining temporal changes in older adults’ life circumstances and the services granted at the *point of LTC entry.* These patterns may reflect shifts in the selection mechanisms governing entry into LTC. We further investigated factors associated with “late entry” into LTC, defined as admission with a high level of dependency. These findings are particularly relevant for policy development and service planning, as they can inform strategies to enhance service targeting and support preventive efforts aimed at avoiding or delaying the need for formal care.

To contextualise our analyses and delineate the parameters of our discussion, we revisit the conceptual framework (Fig. 1), which positions entry into LTC within the broader context of the care-seeking process. In interpreting our findings, it is important to highlight that although access to Sweden’s universal needs-based LTC system is formally guaranteed, the presence of care needs (Box A) does not automatically translate into utilisation (Box D). Rather, the decision to seek LTC (Box B) is shaped by individual predisposing and enabling factors (Box E), as well as environmental factors (Box F). The effect of these on the pathway from care need to LTC utilisation can further be assumed to be shaped by broader structural conditions, such as welfare arrangements, social norms, municipal guidelines and cost-containment strategies (Fig. 1).

### Older adults increasingly entered LTC with extensive care needs

In line with the study’s first objective, we found that while sociodemographic characteristics and informal care access remained relatively stable between 2015 and 2022, the proportion of individuals entering LTC with extensive ADL needs increased from 21 to 29%. Several developments could potentially help explain this key finding. As illustrated in Fig. 1, one possible explanation may be an increase in the incidence of suddenly occurring extensive care needs. Although functional decline typically progresses gradually with age, it can also occur abruptly due to acute medical events. Yet, national register data do not indicate an increase in conditions commonly associated with sudden severe functional decline, such as strokes or fractures (National Board of Health and Welfare 2024), thereby challenging this explanation.

Another potential explanation for the observed increase in older adults delaying LTC entry until care needs become extensive is a shift in local policies, involving stricter needs assessments that may exclude individuals with less severe needs from accessing LTC. However, outright rejections of home care applications remain rare, and most denials concern the frequency of assistance with specific tasks, such as showering or walking. In 2022, only 0.8% of municipal decisions regarding home care were rejections (City of Stockholm 2023). Moreover, given current guidelines emphasizing timely support to prevent further deterioration, and the availability of practical assistance even for individuals whose needs are limited to household tasks, it is unlikely that stricter eligibility criteria alone explain the observed increase in the proportion of older adults entering LTC with such extensive care needs that they require assistance with 4–5 personal ADLs. Another structural factor potentially delaying LTC requests is the availability of tax deductions for privately purchased household services. Among Stockholm residents aged ≥ 65, usage increased from 16% in 2015 to 25% in 2022 (Statistics Sweden 2024). While this may partly explain reduced LTC entry among those without personal ADL needs, it does not account for the rise in individuals requiring extensive assistance, as privately purchased personal care remains virtually non-existent. The COVID-19 pandemic introduces an additional structural dimension that may have shaped individual attitudes and beliefs. Though not examined in this study, it could plausibly have contributed to reduced care-seeking. Notably, while the proportion of individuals entering LTC with extensive care needs was already rising between 2015 and 2018, this trend accelerated from 2019 to 2022. Increased media focus on LTC quality issues and retrenchment (Giritli Nygren et al. 2021) may have further deterred those with less severe needs.

### Factors associated with late entry into LTC when care needs are already extensive

In line with the second objective, findings indicate that cohabiting older adults and those receiving informal care at least weekly were more likely to enter LTC with extensive ADL dependencies and cognitive impairment. In contrast, living with a partner already using LTC reduced the likelihood of entering home care at a late stage. As illustrated in Fig. 1, systems design and cultural norms shape how families manage informal caregiving, initiate formal care requests, and under what conditions care is provided within different welfare regimes. Nevertheless, previous research generally shows that family support tends to reduce the use of formal LTC, despite cross-national variation (Artamonova et al. 2023, Bonsang 2009, Geerts and Van den Bosch 2012, Haberkern and Szydlik 2010). However, comparisons across studies and countries are complicated by differences in study design and definitions of informal care. Although Sweden offers universal, needs-based LTC with limited legal obligations for families (Geerts and Van den Bosch 2012), family caregiving remains a central component (National Board of Health and Welfare 2021; Szebehely and Meagher 2018; Wimo et al. 2020). Partners typically provide more extensive care, while adult children often assist with practical tasks and social support (Ulmanen and Szebehely 2015; von Saenger et al. 2023). Relatives, especially adult children, often initiate LTC applications, and informal and formal LTC frequently overlap (Courbage et al. 2023; Johansson et al. 2018; Sperber et al. 2014). Our finding that individuals living with a partner already receiving care are less likely to enter long-term care late may reflect earlier contact with municipal needs assessors and home care staff, thereby facilitating timelier access to services.

Welfare state arrangements, see Fig. 1, also influence the role of individual or family financial resources in LTC access. Although Sweden’s system is designed to ensure access based on need, our results show that individuals with lower income were more likely to enter LTC with extensive care needs. This may suggest delayed application due to perceived financial barriers, despite low, income-related fees and available exemptions. The lack of income-related differences in service levels once LTC is granted supports the interpretation that perceived costs may deter early application, rather than influence service provision. When using severe worry/insecurity as an indicator of extensive care needs, associations with sociodemographic factors and access to informal care were weaker. The finding that individuals in the youngest age group (65–69 years) were more likely to enter LTC care with such needs compared to older age groups may reflect prior engagement with social services and the administrative transition into LTC for older adults that occurs at age 65.

### Type and amount of granted LTC

Aligned with the third objective, our findings show that most individuals entered LTC through home care, consistent with Sweden’s aging-in-place policy. Although assessed care needs, in terms of ADL disabilities, mobility limitations and cognitive impairment, showed the strongest association with the intensity of granted LTC, the amount of home care granted for comparable dependency levels slightly declined over the eight-year period. Direct transitions to institutional care became even less common, reflecting municipal guidelines prioritising home-based services. Within our conceptual framework (Fig. 1), these trends may be linked to shifting local policies, amounting to stricter needs assessments and/or shifts in care-seeking behaviour due to changing attitudes and beliefs. Although aging-in-place has long been a cornerstone of Swedish Eldercare policy, it has increasingly functioned as a key cost-containment strategy, contributing to a significant reduction in institutional care beds. Correspondingly, data from Stockholm indicate a declining likelihood of being granted institutional care between 2015 and 2022, even among home care recipients with extensive needs (Meinow et al. 2020a; von Berens et al. 2025). This may reflect more restrictive eligibility criteria, or a reluctance among older adults to apply, shaped by MNAs who may signal low approval prospects (Söderberg et al. 2014). The slight reduction in home care hours may stem from tighter time allocations for specific tasks (Jordahl et al. 2023) and/or a shift in in care-seeking behaviour, where individuals initially opt for fewer assistance hours. It is also possible that service levels are adjusted shortly after care initiation (Zingmark and Norström 2021).

### Strengths, limitations and future research

A major strength of this study is the use of a unique and comprehensive dataset covering all individuals who entered LTC in Stockholm between 2015 and 2022. Unlike many register-based studies, which are typically restricted to care utilisation, this data also contains information on assessed functional impairments and disabilities as indicators of care needs that form the basis for LTC eligibility assessments, independent of medical diagnoses. This enables a more nuanced analysis of the relationship between care needs and the services granted. Although the study is limited to a single municipality, Stockholm is the largest in Sweden and accounts for approximately 10% of the population aged 65 and older. While the proportion of older adults receiving LTC varies across municipalities, the underlying mechanisms influencing care-seeking behaviour and need assessments are likely to be similar. To the best of our knowledge, no previous Swedish study has utilised a complete register of first-time LTC users that includes both granted services and key indicators of need based on functional impairments and disabilities.

Nevertheless, several limitations should be acknowledged. Because we relied on existing register data, our analyses were restricted to individuals who applied for and were granted LTC, and to a predefined set of variables representing a relatively narrow range of indicators that may influence the association between care needs and LTC use. To better understand *why* older adults increasingly enter LTC only when care needs have become extensive, and to assess whether reduced care provision at a given level of need reflects shifting attitudes and greater reluctance toward LTC, or instead indicates stricter eligibility criteria and narrower time frames, future research should compare the characteristics of those who apply for LTC with those who do not (Box B). In addition, information on attitudes and beliefs about the LTC system (Box E), more detailed data on multiple dimensions of care needs, the social environment (Box F; e.g., the presence of next of kin who coordinate contacts with health and social care and advocate on behalf of the older person), and the physical environment (such as housing accessibility) would be valuable for gaining deeper insight into the factors shaping applications for and approval of LTC. Finally, including individuals whose applications were rejected (Box G) would provide insight into how resources are allocated, given that these individuals had applied.

As with most register-based research, the data used in this study were collected during routine administrative procedures rather than for research purposes, and without systematic attention to inter-rater reliability. Furthermore, the categories used to classify care needs may encompass heterogeneity, which should be considered when interpreting the findings.

Relatedly, while the study enables an analysis of the alignment between assessed needs and granted services, it does not provide information on whether the support received was sufficient to ensure a well-functioning everyday life, in accordance with the rights stipulated in the Social Services Act (Ministry of Health and Social Affairs 2024).

### Implications

This study illustrated that entry into LTC reflects a complex interplay between the emergence of individual care needs, the inclination to seek care, and prevailing policies and eligibility criteria. Our findings suggest that older adults in Stockholm increasingly enter LTC at a stage when care needs are already extensive. This trend may indicate missed opportunities for early interventions in the home that could slow or prevent the escalation of care needs (Facchinetti et al. 2020). It also stands in contrast to current policy ambitions, which emphasize early-stage support aimed at prevention and delaying the onset of severe care needs (National Board of Health and Welfare 2025; Sweden 2025).These findings raise important questions about the factors contributing to delayed LTC entry despite extensive care needs, such as potential shifts in individual attitudes and beliefs concerning formal LTC. Further research is needed to explore why older individuals increasingly postpone entry into LTC, what precipitates their transition, and how they manage daily life prior to entry. Such insights are essential for designing care services that are responsive to evolving user needs and for effectively targeting preventive strategies that align with national policy goals.

## Supplementary Information

Below is the link to the electronic supplementary material.

Supplementary file1 (DOCX 65 KB)

## Data Availability

The datasets generated during and/or analysed during the current study are available from the corresponding author on reasonable request.
