# Supplementary material for: Older people in Sweden increasingly enter long-term care with extensive care needs–a register study of first-time users based on the SNAC Stockholm Eldercare study
Source: Eur J Ageing. 2026 Jan 13;23(1):7. doi: 10.1007/s10433-025-00906-5 (PMC12847603; doi:10.1007/s10433-025-00906-5)
Supplement: Supplementary file 1 — Supplementary file1 (DOCX 65 KB) [file 10433_2025_906_MOESM1_ESM.docx]

**Supplementary tables, S1- S3**

Table S1. Estimated probability of entering LTC with a high level of care needs according to ADL dependencies, cognitive impairment, and problems with worry/insecurity. Bivariate models presented as predictive margins.

|  | 4-5 ADL dependencies | | | | Severe/ very severe cognitive impairment | | | | Severe/ very severe feelings of worry/insecurity | | | |
| --- | --- | --- | --- | --- | --- | --- | --- | --- | --- | --- | --- | --- |
|  | PM^a^ |  | 95 % CI | | PM^a^ |  | 95 % CI | | PM^a^ |  | 95 % CI | |
| Year | | | | | | | | | | | | |
| 2015 (Ref.) | 0.209 |  | 0.194; | 0.222 | 0.103 |  | 0.092; | 0.113 | 0.096 |  | 0.086; | 0.106 |
| 2016 | 0.220 |  | 0.207; | 0.233 | 0.111 |  | 0.101; | 0.121 | 0.104 |  | 0.094; | 0.115 |
| 2017 | 0.229 | ***** | 0.216; | 0.242 | 0.101 |  | 0.091; | 0.110 | 0.102 |  | 0.092; | 0.112 |
| 2018 | 0.238 | ***** | 0.225; | 0.251 | 0.105 |  | 0.095; | 0.114 | 0.106 |  | 0.096; | 0.116 |
| 2019 | 0.231 | ***** | 0.218; | 0.244 | 0.102 |  | 0.093; | 0.112 | 0.098 |  | 0.089; | 0.108 |
| 2020 | 0.272 | ******* | 0.257; | 0.287 | 0.113 |  | 0.103; | 0.124 | 0.108 |  | 0.097; | 0.119 |
| 2021 | 0.287 | ******* | 0.273; | 0.300 | 0.117 |  | 0.107; | 0.127 | 0.106 |  | 0.096; | 0.116 |
| 2022 | 0.289 | ******* | 0.274; | 0.304 | 0.115 |  | 0.105; | 0.126 | 0.106 |  | 0.095; | 0.116 |
| Individual-level and environmental factors | | | | | | | | | | | | |
| Age | | | | | | | | | | | | |
| 65-69 | 0.232 |  | 0.219; | 0.245 | 0.095 | ******* | 0.086; | 0.104 | 0.178 | ******* | 0.165; | 0.190 |
| 70-74 | 0.240 |  | 0.227; | 0.252 | 0.093 | ******* | 0.084; | 0.101 | 0.103 |  | 0.094; | 0.112 |
| 75-79 | 0.233 |  | 0.222; | 0.244 | 0.107 |  | 0.099; | 0.115 | 0.090 |  | 0.083; | 0.098 |
| 80-84 (Ref.) | 0.246 |  | 0.236; | 0.257 | 0.118 |  | 0.110; | 0.126 | 0.093 |  | 0.085; | 0.100 |
| 85-89 | 0.247 |  | 0.236; | 0.258 | 0.120 |  | 0.112; | 0.129 | 0.088 |  | 0.080; | 0.096 |
| 90+ | 0.295 | ******* | 0.280; | 0.309 | 0.109 |  | 0.098; | 0.119 | 0.087 |  | 0.077; | 0.097 |
| Income | | | | | | | | | | | | |
| Q1 | 0.287 | *** | 0.277; | 0.298 | 0.113 | * | 0.105; | 0.121 | 0.145 | ******* | 0.136; | 0.153 |
| Q2 | 0.243 | ******* | 0.233; | 0.252 | 0.103 |  | 0.096; | 0.109 | 0.105 | ******* | 0.098; | 0.113 |
| Q3 | 0.243 | ******* | 0.234; | 0.253 | 0.107 |  | 0.101; | 0.114 | 0.085 | ******* | 0.078; | 0.091 |
| Q4 (Ref.) | 0.217 |  | 0.207; | 0.226 | 0.111 |  | 0.104; | 0.118 | 0.083 |  | 0.076; | 0.089 |
| Gender and cohabitation status | | | | | | | | | | | | |
| Women living alone (Ref.) | 0.186 |  | 0.179; | 0.192 | 0.080 |  | 0.075; | 0.085 | 0.107 |  | 0.101; | 0.112 |
| Men living alone | 0.195 |  | 0.185; | 0.205 | 0.088 |  | 0.081; | 0.095 | 0.090 | ******* | 0.082; | 0.097 |
| Women cohabiting | 0.321 | ******* | 0.308; | 0.335 | 0.154 | ******* | 0.143; | 0.164 | 0.127 | ******* | 0.116; | 0.137 |
| Men cohabiting | 0.383 | ******* | 0.371; | 0.396 | 0.159 | ******* | 0.150; | 0.168 | 0.093 | ****** | 0.085; | 0.100 |
| Frequency of informal care | | | | | | | | | | | | |
| Never/seldom (Ref.) | 0.162 |  | 0.155; | 0.169 | 0.062 |  | 0.057; | 0.066 | 0.093 |  | 0.087; | 0.098 |
| At least once a week | 0.313 | ******* | 0.305; | 0.320 | 0.149 | ******* | 0.143; | 0.155 | 0.113 | ******* | 0.108; | 0.118 |
| Missing | 0.224 | ******* | 0.212; | 0.236 | 0.081 | ******* | 0.072; | 0.089 | 0.095 |  | 0.086; | 0.105 |
| Lives with a partner who is also granted home care | | | | | | | | | | | | |
| No (Ref.) | 0.227 |  | 0.222; | 0.232 | 0.089 |  | 0.085; | 0.092 | 0.090 |  | 0.086; | 0.093 |
| Yes | 0.194 | ******* | 0.179; | 0.209 | 0.082 |  | 0.072; | 0.093 | 0.044 | ******* | 0.036; | 0.053 |
| Missing ^b^ | 0.695 | ******* | 0.671; | 0.718 | 0.509 | ******* | 0.483; | 0.535 | 0.448 | ******* | 0.421 | 0.475 |
| Born in a Nordic country | | | | |  |  |  |  |  |  |  |  |
| No (Ref.) | 0.277 |  | 0.260; | 0.294 | 0.135 |  | 0.122; | 0.148 | 0.162 |  | 0.147; | 0.176 |
| Yes | 0.233 | ******* | 0.226; | 0.240 | 0.106 | ******* | 0.101; | 0.111 | 0.097 | ******* | 0.092; | 0.102 |
| Missing | 0.258 | ***** | 0.250; | 0.266 | 0.106 | ******* | 0.100; | 0.111 | 0.098 | ******* | 0.092; | 0.103 |

^a^ Predictive margin (estimated marginal probabilities) Ref.= Reference category. ^b^ Information is not available for individuals granted institutional care. For individuals granted home care services, data is missing for n = 86. Significance levels are based on average marginal effect tests. * p≤0.05 ** p≤0.01 *** p≤0.001

Table S2. Estimated probability of being granted residential care instead of home care by year, individual-level and environmental factors and assessed care needs. Bivariate model presented as predictive margins.

|  | Bivariate Model | | | |
| --- | --- | --- | --- | --- |
|  | PM^a^ |  | 95% CI | |
| Year | | | | |
| 2015 (Ref.) | 0.052 |  | 0.045; | 0.059 |
| 2016 | 0.056 |  | 0.049; | 0.063 |
| 2017 | 0.050 |  | 0.043; | 0.057 |
| 2018 | 0.056 |  | 0.049; | 0.063 |
| 2019 | 0.049 |  | 0.043; | 0.056 |
| 2020 | 0.068 | ****** | 0.060; | 0.077 |
| 2021 | 0.067 | ****** | 0.059; | 0.075 |
| 2022 | 0.055 |  | 0.048; | 0.063 |
| Individual-level and environmental factors | | | | |
| Age | | | | |
| 65-69 | 0.073 | ******* | 0.065; | 0.081 |
| 70-79 | 0.038 | ******* | 0.035; | 0.042 |
| 80-89 (Ref.) | 0.054 |  | 0.050; | 0.058 |
| 90+ | 0.098 | ******* | 0.089; | 0.108 |
| Gender and cohabitation status | | | | |
| Women living alone (Ref.) | 0.054 |  | 0.050; | 0.058 |
| Men living alone | 0.064 | ****** | 0.058; | 0.070 |
| Women cohabiting | 0.036 | ******* | 0.030; | 0.041 |
| Men cohabiting | 0.042 | ******* | 0.036; | 0.047 |
| Income | | | | |
| Quartile 1 (Ref.) | 0.087 |  | 0.080; | 0.093 |
| Quartile 2 | 0.048 | ******* | 0.043; | 0.052 |
| Quartile 3 | 0.049 | ******* | 0.044; | 0.053 |
| Quartile 4 | 0.046 | ******* | 0.041; | 0.050 |
| Frequency of informal care | | | | |
| Never/seldom (Ref.) | 0.047 |  | 0.043; | 0.051 |
| At least once a week | 0.046 |  | 0.043; | 0.049 |
| Missing | 0.101 | ******* | 0.094; | 0.109 |
| Born in a Nordic country | | | | |
| No (Ref.) | 0.073 |  | 0.063; | 0.083 |
| Yes | 0.055 | ****** | 0.051; | 0.059 |
| Missing | 0.055 | ****** | 0.051; | 0.059 |
| Assessed care needs | | | | |
| Number of ADL-dependencies | | | | |
| 0 (Ref.) | 0.007 |  | 0.006; | 0.009 |
| 1 | 0.015 | ******* | 0.011; | 0.019 |
| 2 | 0.042 | ******* | 0.035; | 0.049 |
| 3 | 0.092 | ******* | 0.078; | 0.105 |
| 4 | 0.074 | ******* | 0.066; | 0.083 |
| 5 | 0.293 | ******* | 0.275; | 0.311 |
| Missing | 0.105 | ******* | 0.093; | 0.116 |
| Mobility limitations | | | | |
| None/slight (Ref.) | 0.027 |  | 0.025; | 0.029 |
| Moderate | 0.046 | ******* | 0.041; | 0.051 |
| Severe | 0.216 | ******* | 0.201; | 0.231 |
| Missing | 0.139 | ******* | 0.123; | 0.155 |
| Cognitive impairment | | | | |
| None (Ref.) | 0.017 |  | 0.015; | 0.019 |
| Slight | 0.056 | ******* | 0.051; | 0.062 |
| Severe/ very severe | 0.312 | ******* | 0.293; | 0.331 |
| Missing | 0.095 | ******* | 0.085 | 0.105 |
| Feelings of worry/insecurity | | | | |
| None (Ref.) | 0.018 |  | 0.016; | 0.020 |
| Slight | 0.070 | ******* | 0.063; | 0.076 |
| Severe/ very severe | 0.259 | ******* | 0.241; | 0.277 |
| Missing | 0.075 | ******* | 0.068; | 0.082 |

^a^ Predictive margin (estimated marginal probabilities) Ref.= reference category. Significance levels are based on average marginal effect tests. * p≤0.05 ** p≤0.01 *** p≤0.001

Table S3. Predicted monthly hours of home care (predicted margins), by year, individual-level and environmental factors and assessed care needs. Bivariate model.

|  | Bivariate model | | | |
| --- | --- | --- | --- | --- |
|  | PM^a^ |  | 95% CI | |
| Year | | | | |
| 2015 (Ref.) | 24.85 |  | 24.69; | 25.02 |
| 2016 | 24.43 | ******* | 24.27; | 24.59 |
| 2017 | 25.60 | ******* | 25.44; | 25.76 |
| 2018 | 25.29 | ******* | 25.13; | 25.45 |
| 2019 | 24.52 | ******* | 24.36; | 24.67 |
| 2020 | 26.18 | ******* | 26.01; | 26.36 |
| 2021 | 26.59 | ******* | 26.42; | 26.75 |
| 2022 | 26.33 | ******* | 26.16; | 26.51 |
| Individual-level and environmental factors | | | | |
| Age | | | | |
| 65-69 | 26.55 | *** | 26.39; | 26.72 |
| 70-79 | 25.11 | ******* | 25.01; | 25.21 |
| 80-89 (Ref.) | 24.85 |  | 24.76; | 24.94 |
| 90+ | 27.42 | ******* | 27.24; | 27.60 |
| Gender and cohabitation status | | | | |
| Women living alone (Ref.) | 24.30 |  | 24.21; | 24.38 |
| Men living alone | 24.49 | ****** | 24.37; | 24.61 |
| Women cohabiting | 26.38 | ******* | 26.22; | 26.53 |
| Men cohabiting | 29.36 | ******* | 29.22; | 29.51 |
| Income | | | | |
| Quartile 1 (Ref.) | 27.54 |  | 27.41; | 27.67 |
| Quartile 2 | 24.88 | ******* | 24.76; | 24.99 |
| Quartile 3 | 24.76 | ******* | 24.65; | 24.88 |
| Quartile 4 | 24.89 | ******* | 24.77; | 25.00 |
| Frequency of informal care | | | | |
| Never/seldom (Ref.) | 21.81 |  | 21.72; | 21.90 |
| At least once a week | 29.03 | ******* | 28.94; | 29.12 |
| Missing | 23.23 | ******* | 23.10; | 23.36 |
| Lives with a partner who is also granted home care | | | | |
| No (Ref.) | 25.93 |  | 25.87; | 25.99 |
| Yes | 21.12 | *** | 20.95; | 21.29 |
| Born in a Nordic country | | | | |
| No (Ref.) | 27.94 |  | 27.74; | 28.15 |
| Yes | 24.81 | *** | 24.73; | 24.89 |
| Missing | 25.72 | *** | 25.63; | 25.81 |
| Assessed care needs | | | | |
| Number of ADL dependencies | | | | |
| 0 (Ref.) | 9.74 |  | 9.68; | 9.79 |
| 1 | 18.97 | ******* | 18.83; | 19.11 |
| 2 | 31.03 | ******* | 30.82; | 31.23 |
| 3 | 40.74 | ******* | 40.44; | 41.05 |
| 4 | 58.72 | ******* | 58.47; | 58.97 |
| 5 | 71.17 | ******* | 70.78; | 71.57 |
| Missing | 21.66 | ******* | 21.47; | 21.84 |
| Mobility limitations | | | | |
| None/slight (Ref.) | 16.32 |  | 16.26; | 16.37 |
| Moderate | 35.54 | ******* | 35.39; | 35.68 |
| Severe | 71.48 | ******* | 71.14; | 71.82 |
| Missing | 21.15 | ******* | 19.92; | 20.37 |
| Cognitive impairment | | | | |
| None (Ref.) | 23.37 |  | 23.30; | 23.44 |
| Slight | 29.64 | ******* | 29.51; | 29.77 |
| Severe/ very severe | 34.73 | ******* | 34.44; | 35.02 |
| Missing | 25.06 | ******* | 23.88; | 24.23 |
| Feelings of worry/insecurity | | | | |
| None (Ref.) | 22.80 |  | 22.73; | 22.88 |
| Slight | 29.97 | ******* | 29.82; | 30.11 |
| Severe/very severe | 33.09 | ******* | 32.82; | 33.37 |
| Missing | 26.52 | ******* | 26.38; | 26.67 |

^a^ Predictive margin (predicted average hours/month) Ref.= Reference category. Significance levels are based on average marginal effect tests. * p≤0.05 ** p≤0.01 *** p≤0.001
